# Toxic trajectories under future climate conditions

**DOI:** 10.1371/journal.pone.0226958

**Published:** 2019-12-23

**Authors:** Richard A. Marcantonio, Sean Field, Patrick M. Regan

**Affiliations:** 1 The Kroc Institute for International Peace Studies and the Anthropology Department, University of Notre Dame, Notre Dame, IN, United States of America; 2 The Anthropology Department, the University of Notre Dame, Notre Dame, IN, United States of America; 3 The Kroc Institute for International Peace Studies and the Political Science Department, University of Notre Dame, Notre Dame, IN, United States of America; University of Vigo, SPAIN

## Abstract

Extreme weather events, driven by changing climatic conditions, interact with our built environment by distributing—or redistributing—environmental risk and damaging physical infrastructure. We focus on the role of extreme weather events in the distribution of toxic substances within and between residential communities in the largest cities in the United States (US). We explore the impact of projected inland and coastal flooding on the redistribution of toxicity from known contaminated sites, and how patterns of toxic flow change the total population and social demographics of the population at risk from toxic materials. We use the Urban Adaptation Assessment and data on toxic site locations from the US government to evaluate risk of toxin dispersion from flooding in cities and down to the census tract level for the period 2021–2061. We demonstrate that future climate conditions significantly increase the risk of the dispersion of toxins from contaminated sites by 2041.

## Introduction

Extreme weather events, driven by changing climatic conditions, interact with our built environment by distributing—or redistributing—environmental risk and damaging physical infrastructure [1–3]. We focus on the role of extreme weather events in the distribution of toxic substances within and between residential communities in the United States’ (US) largest cities.

Industrial and military processes generate toxic materials contaminating over 900,000 hazardous waste sites across the US. Most of the toxins have long half-lives, some for thousands of years [4], meaning that toxins must either be actively remediated or stored to avoid ground, water, or human contamination. Currently, approximately three percent of hazardous waste sites are enrolled in federally funded remediation or containment programs, while the vast majority of sites are not being actively monitored. Under normal conditions, the toxins located at all hazardous waste sites could be considered localized problems, posing risk only if contaminants move beyond containment sites. Yet, current extreme weather events have demonstrated that localized problems can be redistributed, thereby impacting larger regions [2,5]. Moreover, future predictions suggest that extreme weather events will become more frequent and intense [6–10].

Previous research has worked to understand the relationship between flooding and the spread of contaminants [11]. However, we suggest that these efforts underestimate the potential risk of contaminates being redistributed by flood for several reasons. First, some of the most widely utilized flood estimates, produced by the Federal Emergency Management Agency (FEMA), have been demonstrated to underestimate future flood risks because they do not consider the impacts of climate change [10,12]. Additionally, FEMA maps are heavily influence by local politics, where local officials can choose whether certain areas are listed as a flood zone or not, due to requisite insurance liabilities associated with flood zones [12]. Second, most research employs Superfund sites as the unit of analysis representing contaminated sites, limiting their list to less than approximately 2,000 contaminated sites [13–16]. Simply including toxic sites beyond Superfund designation—i.e. including subsets of Resource Conservation and Recovery Act Corrective Action (RCRA CA) and Brownfield sites that can be as, or more, contaminated than Superfund sites—increases the number of considered toxic sites by a factor of 15, highlighting a critical underestimation in previous studies on flooding and contamination risks.

Additionally, the aggregate risk of flood is often misunderstood (citation). A 1% annual risk of flooding reflects seemingly small and independent probabilities yet generates a 25% chance of experiencing a flood over a 20-year period and a 33% chance over 30 years. The risk is much more consequential than annual projections would suggest, and climate change portends to increase expected flood frequency and range. We demonstrate that future climate conditions significantly alter these risk calculations and the concomitant risk of toxic material dispersion due to flooding. Understanding the dispersion of toxins from flooding under different potential climate conditions—a rapidly increasing, but minimally accounted for global phenomenon and risk (citation)—is essential for effective environmental policy and climate adaptation decision-making [17].

To address these issues, we assess the impact of flooding on the redistribution of toxins from known contaminated sites, using more robust measures of flooding and contaminated sites across the United States (US). Specifically, we explore the impact of extreme weather events, via projected inland and coastal flooding, on the redistribution of toxins from known contaminated sites, and how patterns of toxic flow change the social demographics at risk from toxic materials. We use the Urban Adaptation Assessment (UAA) flood risk and demographic data, which includes all US cities with populations greater than 100,000 (N = 274), and data on toxic site locations from the US government (including Superfund, Brownfields and RCRA CA sites; N = 29,444) to evaluate risk of toxin dispersion from flood events for the period 2021–2061 [18–24]. To ground these projections, we employ two empirical case studies—Hurricanes Harvey and Sandy—to demonstrate how recent flood events have redistributed toxins from contaminated sites to surrounding urban areas. First, we briefly review the steps taken to structure our analyses.

## Methods

Our core question is how many contaminated sites across the US are at risk of flooding in future climate conditions. Therefore, we began with flood projections that were produced by the UAA, which show the probability of annual flood events for 274 US cities (comprised of 25,569 census tracts) between 2021–2065. The UAA defines a flood event as six consecutive days of precipitation above the city’s 90^th^ percentile based on historic trends (1950–99). The UAA annual flood estimates are derived from National Oceanic and Atmospheric Association (NOAA) projections. The NOAA projections are based on the conservative Representative Concentration Pathway (RCP) 4.5 ensemble from the 2014 International Panel on Climate Change (IPCC) report [25]. While we assert that FEMA assessed flood probabilities underestimate the likelihood of future flooding, the UAA relied on FEMA flood zoning for 1-in-100-year floods to determine populations living in high risk flood zones within census tracts as it is the best available measure. In sum, the UAA precipitation-based measure of flooding is used to determine future flood probabilities based on NOAA precipitation projections; FEMA flood zoning indicates what areas within UAA included cities are likely to be flooded. We relied on both measures for our projections on how flooding, contaminated sites, and people in high risk flood zones interact. We summarized these data by calculating the total number of cities that had a forecast greater than their baseline of flood for each year. These data do not necessarily forecast the severity or spatial extent of floods in each city, but rather tell us which cities are expected to flood annually—i.e. the distribution of cities flooded rather than the distribution of flooding within cities. Our forecasts focus on flooding due to high precipitation events; however, we also consider climate-driven increases in risk of flooding due to sea-level rise and storm surge.

### Mapping relationships between floods and contaminated sites

Using the tabulated UAA city flood values, we worked to measure the relationship between projected flooding events and contaminated sites within UAA city limits across time. First, we downloaded location data on toxic sites from *Cleanups in My Community Map* (CIMC), a national register updated by the US Environmental Protection Agency (EPA) which documents contaminated sites in the US that are either being actively remediated or monitored through an EPA proctored program [18]. From this register, we selected Superfund, RCRA Corrective Action, and Brownfield sites that were being actively remediated or under forced corrective action, resulting in the location of 29,444 contaminated sites within the boundaries of the US. Next, we ran a series of spatial queries in the statistical software program R to identify the number of contaminated sites that are within the boundaries of any UAA city that had a probability of flooding in the decade intervals from 2021-2061.This analysis produced the number and location of all contaminated sites in cities that are expected to flood in 2021, 2031, 2041, 2051, and 2061. Further, using UAA demographic data which is recorded at the census-tract level, we were able to tabulate demographic information regarding populations living in a census tract with a contaminated site that is projected to flood. Specifically, we calculated 1) total populations; 2) populations in FEMA designated high risk flood zones, and; 3) average median household income for each census tract with a contaminated site. To understand the potential impact of moving contaminants, we expanded our analysis to measure all census tracts that lie within a thousand meters of the edge of any census tracts that had both; 1) a contaminated site within its boundary, and 2) a greater than zero percent chance of flooding in the years 2021, 2031, 2041, 2051, and 2061.

### Mapping case studies

To demonstrate the movement of toxins in case study scenarios we measured the number of contaminated sites inundated during Hurricane Harvey in Houston in 2017, and during Hurricane Sandy in New York in 2012.

The extent of inundation during Hurricane Harvey was taken from multiple shapefile sources—FEMA and the USGS Texas Water Science Center (TXWSC)–and merged to document the total area of inundation in Harris county (the main portion of Houston city) and multiple counties to the North and West of Houston. We then ran a similar set of spatial queries as used in the prior analyses, to understand how many of the total number of contaminated sites within Houston city limits (N = 114) were directly inundated during Hurricane Harvey and queried associated demographic data. Our results were mapped using QGIS Version 3.8, with satellite imagery and a ten-meter digital elevation model (DEM) developed by the USGS [26].

Inundation extents during Hurricane Sandy [27] were used to calculate how many of the total number of contaminated sites within New York city limits (N = 59) were flooded during the storm. As before, demographic data was also tabulated. These results were also mapped using QGIS Version 3.8, with satellite imagery and a one-meter DEM made available by the NYC Open Data repository [28].

All data and scripts necessary for analyses replication can be accessed at our GitHub repository [29].

## Results and findings

### Future flooding

Inland flooding can result from excess precipitation or sea-level rise and storm surges. These two mechanisms point to different trajectories (i.e. downstream vs. upstream flow) for toxic dispersion from inland flooding. Precipitation generated flooding will follow the topography of a city and water will generally flow from higher-to-lower elevations. Toxic sites in the pathway of these flow patterns will be more susceptible to the dispersion of contaminants, which will follow the natural flow of the drainage system. Coastal inundation, on the other hand, also results from sea-level rise and storm surge, both affected by climate change. Inundation from coastal flooding would follow a trajectory different from precipitation-based flooding, following the push of the sea inland, potentially up to and over physical barriers. Toxic sites in the pathway of flooding coming from sea-to-shore will disperse contaminants upstream to the extent that barriers permit. Splash erosion from rainfall, which when combined with a small amount of surface flow, can also quickly entrain and transport eroded contaminants, even if a site is not fully inundated. In short, toxic flows will generally be downstream, but in some areas—namely coastal areas—there is the potential for upstream flow, and a contaminated site does not have to be fully inundated to contribute toxic materials to flood waters.

We define ‘high-risk of flood’ as any city with an increased risk of flooding that is above their baseline in future climate conditions-i.e. an event that would be considered an extreme or major flood event. Using this specification, designated high-risk flood zones within cities with increased flood risk in future conditions exceed their baseline 1% annual chance of flooding, which translates into a greater than 1-in-4 chance (>25%) that area will experience major flooding by 2040, and a greater than 1-in-3 chance (>33%) of major flooding by 2050.

**Fig 1:**
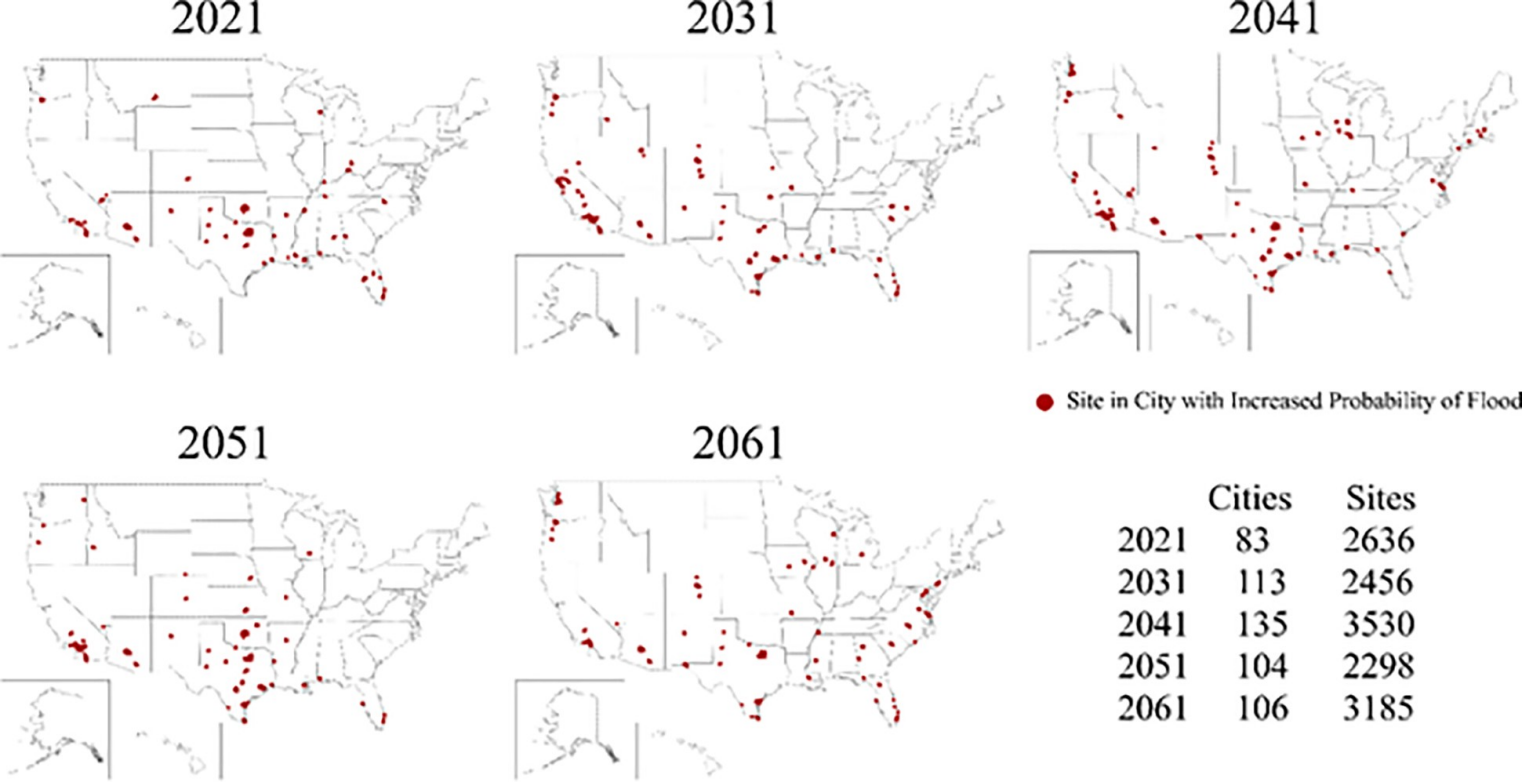
Cities with predicted flood probabilities above their historical baseline for the years 2021, 2031, 2041, 2051, and 2061. The number of sites at risk in each city varies resulting in the possibility of fewer cities, but more sites at risk of flooding—and vice versa—in a given year.

The UAA projections indicate that over the next forty years there will be an increase in the total number of UAA cities flooded annually. The maps in Fig 1 show which UAA cities are at high-risk of flood in the years 2021, 2031, 2041, 2051, and 2061: it is a snapshot of what future flooding might look like given changing climatic conditions. Importantly, over time the distribution of flood events will vary across the entire US and not be isolated to a few regions or areas, suggesting that all cities are to some extent vulnerable to flood risk in the context of future climatic conditions and no region is ‘safe’ or insulated from future climate conditions.

### Flooding, contamination, and at-risk populations

Consistent with the increasing probability of UAA cities experiencing floods is the increased probability of contaminated sites at-risk of flooding each year. Our results show that an increasing number of contaminated sites—be they Superfund, RCRA CA, or Brownfield—are at-risk of flooding between 2021 to 2061, and that the number of people at-risk of contamination by virtue of living in a flood zone in the immediate area of a site also increases over time (see supplemental materials for results for 2021–2061).

For example, in 2021 our results (Table 1) project there are 712 UAA-city census tracts at high-risk of flood, containing 2,636 contaminated sites within their borders. Within these 712 census tracts, there are 2,777,081 people of whom 246,461 are living in high-risk flood zones. The median household income is $39,873—about $20,000 below the national average [30]. Two decades later, in 2041, our analysis indicates there are 3,530 toxic sites at high risk of flood contained within 1,149 census tracts, an increase of 34% in total sites and 61% in total census tracts. Within these census tracts there are 4,910,877 people of whom 326,534 live in a high-risk flood zones, again an increase of 76% and 32% respectively. The median household income in these at-risk zones increases by only 4% to $42,570. In sum, hundreds of thousands of people are at high-risk of flood and contamination spread, and these people at greatest risk tend to be below average income earners.

**Table 1:** Results for analysis of UAA cities, flood risk, and contaminated sites for 2021 and 2041.

Tracts with contaminated sites	2021	2041
Sites in UAA cities with high flood probability	2,636	3,530
# of census tracts with a site in its boundary	712	1149
Total Population	2,777,081	4,910,877
Population in High Risk Flood Zones	246,461	326,534
Average of Median Household Income	39,873	43,293
**Neighbors of tracts with contaminated sites**	**2021**	**2041**
Tracts adjacent to a tract that has a site in its boundary	2,638	5,832
Total Population	11,248,947	25,693,311
Population in High Risk Flood Zone	883,287	1,302,971
Average of Median Household Income	48,591	51,712

Note all values are in 2017 dollars (USD). Population values are from 2017 and are held constant for future projections.

The increase in the number of contaminated sites at-risk of flooding across time and the corresponding population at-risk from flood-based dispersion of contaminants is significant, even though our results are derived from ‘best case scenario’ future climate conditions. Our results are a potential baseline of the risk of contamination from flooding with the potential for significant increases as other variables change over time.

However, contaminants entrained in flood waters can be transported significant distances from their origins. To account for this potential, we expand our analysis of people at-risk of contamination during flood events to include populations that neighbor a census track with at least one contaminated site in its boundaries. This approach undercounts the dispersion of risk but allows us to demonstrate the impact of floods on toxic dispersion. Using our 2021 frame of reference (Table 1), there are potentially 2,638 census tracts that neighbor a tract with a contaminated site that is at high-risk of flooding, tripling the number of tracts at risk—i.e. extending from 712 tracts with at-risk sites to include 2,638 neighboring tracts for a total of 3,350 tracts with or near at risk sites—and increasing the population by a factor of four. The median household income increases by 20% to $48,591 when we include neighboring tracts, highlighting that it is not only lower socioeconomic households at risk of contamination from flooding but also higher socioeconomic areas—i.e. areas that have not had the risk of living near a Superfund site included in the price of the property they live on and a demographic often perceived to be less vulnerable to environmental risk. Because of an increased risk of flood events, in 2041, we estimate that there are an additional 5,832 census tracts would be at high-risk of flood (a 265% increase from ‘tracts with contaminated sites only’ during the same year) which would increase the population living in a flood plain increasing by a factor of four (1,302,971). The flood-dispersed toxins put at risk a wealthier demographic, with a household income of $51,712 (a 19% increase from 2041 without neighbors; a 29% increase on 2021 without neighbors). Including neighboring tracts and future flood projections demonstrates that there is the potential for a major shift in the total population at-risk of contamination from toxic flows from flooding and that the distribution of risk shifts to include wealthier demographics.

### Local level empirical analysis

The risk of toxic dispersal due to flooding has been evidenced at local scales. Superfund sites in New York and Houston released toxic materials as a result of hurricanes Sandy and Harvey [14,15,31–34]. Our analysis suggests that future implications may be more severe and more frequent. These storm events are outliers under contemporary climate conditions; yet, similar extreme weather events have increased in frequency across the US as a result of climate change [35]. More recent events, such as Tropical Storm Imelda that dropped as much rain in parts of Houston as did Hurricane Harvey, are shifting the baseline precipitation distribution of cities such that these storms may eventually cease to be considered outliers (citation). To better demonstrate the implications of our national-level estimates, we use Hurricanes Harvey and Sandy as two case examples of toxic site inundation and the potential for contamination spreading.

#### Hurricane Harvey in Houston

Hurricane Harvey generated a 4 to 10 ft storm surge, and up to 60 inches of rain along the Texas Gulf Coast, flooding some 300,000 structures and destroying over 500,000 cars [36]. It rained for four days straight, inundating inland areas of Houston. Houston received a record setting 48.2 inches of rain over a six-day period, an amount matching their historical annual average of 49.8 inches [37]. Harvey is estimated to have been a 1-in-1,000 year—or a .1% probability—given Houston’s historical rainfall record [38]. Hurricanes Florence, Maria, and Michael, and most recently Hurricane Dorian and Tropical Storm Imelda, all of which set rainfall and other records, all occurred just in the last few years in the Gulf Coast region, suggesting that Harvey, in time, may not be considered an outlier event. We use Harvey to describe the potential risk from contaminated site inundation resulting from flood events in 2041.

**Fig 2:**
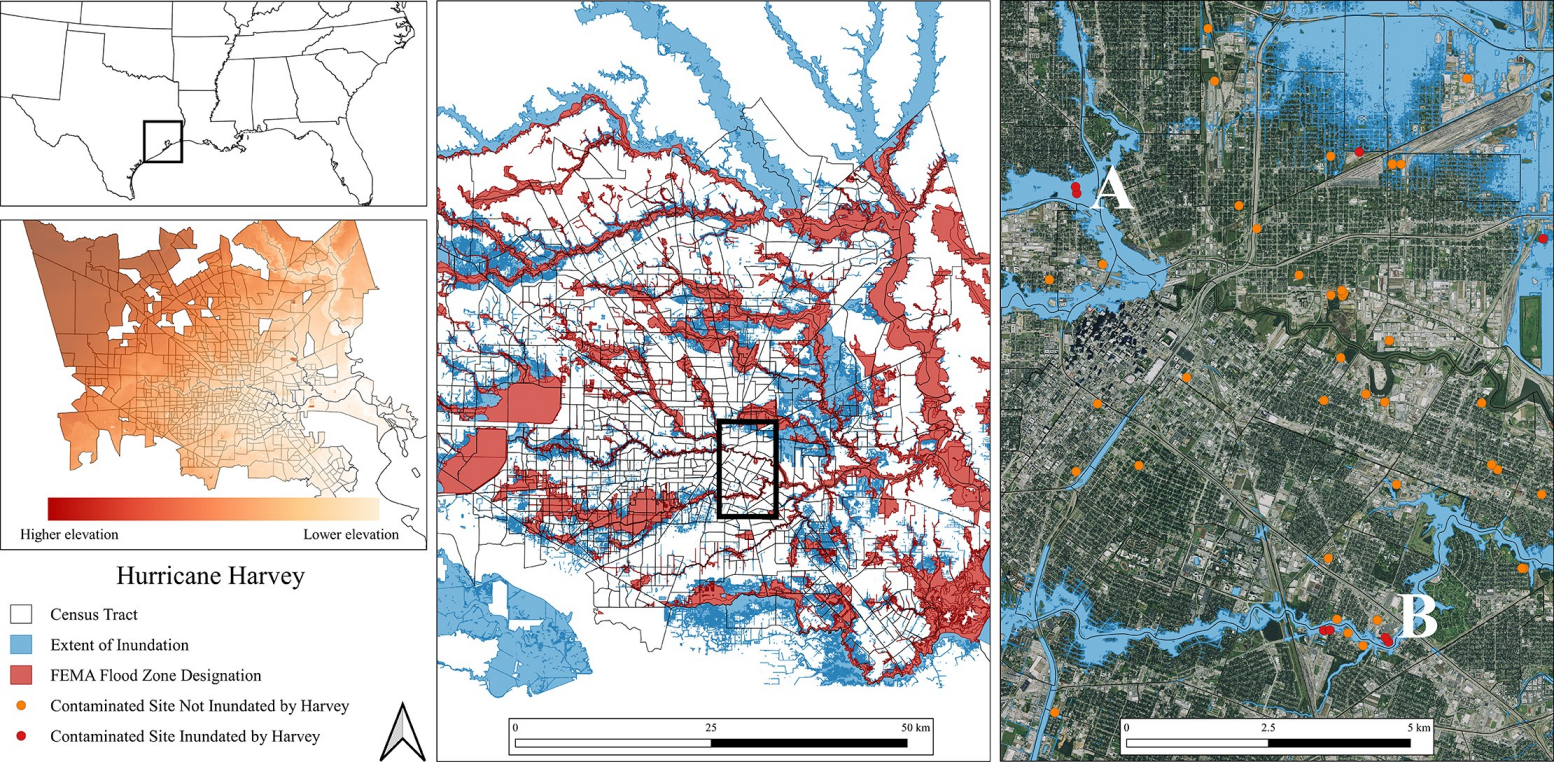
Flooding and inundated contaminated sites during and post-Hurricane Harvey in Houston, Texas.

Fig 2 shows the inundation that occurred in Houston during Harvey and demarcates the distribution of contaminated sites, indicating those that were flooded and those that were not. Our mapping focuses on site flooding and dispersal. Once contaminants are in flood waters, we expect that they will be deposited downstream. Analyses conducted post-Harvey demonstrate that toxins were transported from toxic sites, contaminating surrounding land, communities, and structures [14,31]. For example, the Manchester neighborhood in the East End of Houston—a low socioeconomic neighborhood located next to a contaminated petrochemical refinery that is included in our site list and that was flooded during Harvey—had higher levels of polycyclic aromatic hydrocarbons immediately post-Harvey than pre-Harvey, with flood waters from Harvey determined to be the source of the increase [14]. Since most of the flooding in Houston was due to intense rainfall, entrained contaminants would have flowed downstream in a generally northwest to east-southeast direction due to the topography of the city, contaminating areas outside of expected containment facilities and into areas with demographics that do not reflect the known risk from toxic site locations. On the map on the right-side of Fig 2, point A represents Brownfield sites demonstrated to contain pollutants such as lead and petroleum hydrocarbons and point B marks Brownfield sites demonstrated to contain lead, VOCs, SVOCs, asbestos, and petroleum hydrocarbons [18]. Flood waters from point A travelled directly into downtown Houston, while flood waters from point B flowed into the Gulfgate/Pine Valley, Lawn Dale/Wayside, and Greater East End neighborhoods, with all of these waters flowing into Buffalo Bayou, which saw its waters expand well past its banks and deposit materials along its entire corridor. Neither the sites at point A nor point B have protective measures in-place to protect them from flood waters. Previous estimates are that 13 contaminated sites were flooded during Harvey; however, using our more inclusive dataset we estimate that 26 sites were inundated by flood waters.

#### Hurricane Sandy in New York City

**Fig 3:**
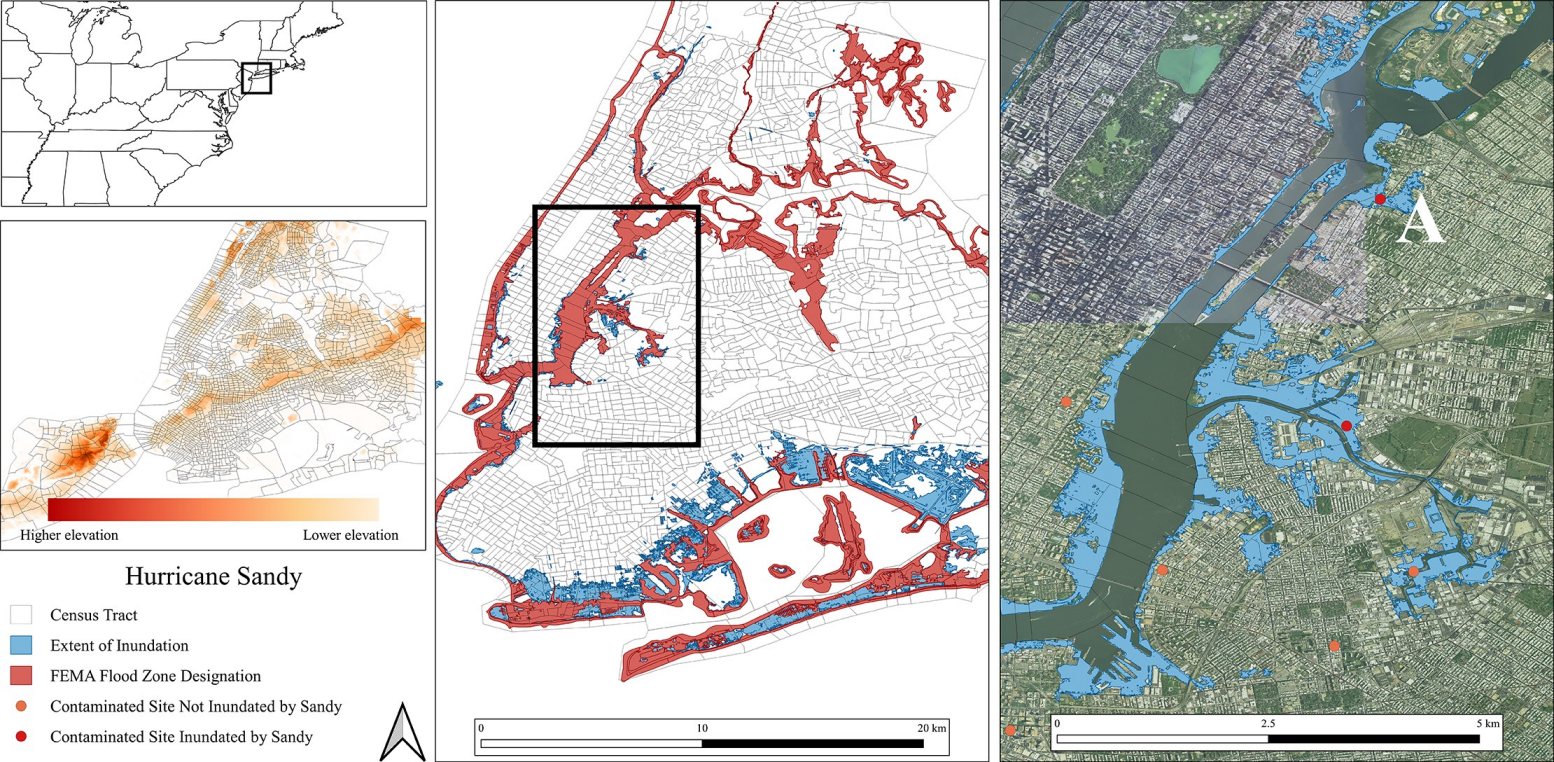
Flooding and inundated contaminated sites that occurred during and post-Hurricane Sandy in New York City, New York.

Unlike Hurricane Harvey, much of the flooding in New York City (NYC) during Hurricane Sandy came from storm surge as opposed to extreme rainfall, though both were instrumental. This means that rather than considering the impact of downstream dispersal, inland movement of water becomes more dominant, particularly given that the location of many contaminated sites are immediately along coast (Fig 3).

In NYC, during Sandy, our analysis shows that eight contaminated sites were inundated and other research conducted post-Sandy demonstrates that contaminants were moved from sites and disbursed into surrounding areas [15,32]. For example, soil samples taken from neighborhoods that were immediately downstream of the flooded Newton Creek and Gowanus Canal Superfund sites, such as in South Side, New York, showed high concentrations of PCB contamination, along with multiple heavy metal toxins [15]. This spike in contamination immediately following Sandy demonstrates the movement of toxins from these sites to the surrounding neighborhoods. When looking at the topography and inundation shown in Fig 3, it is likely that toxins from contaminated sites other than the two Superfund sites previously discussed were moved upland. For example, Point A marks the Nelson Foundry Brownfield site that, despite having EPA cleanup measures taken in the 1990’s, contains highly contaminated soils that require further assessment and remediation, yet they remain unprotected to flood waters currently. Storm surge during Sandy inundated the Foundry and pushed inland almost half a mile, flooding residences, a high school, and many other structures.

Localized *ex ante* and *ex poste* studies of the areas surrounding flooded contaminated sites are necessary to confirm whether toxins have moved or not. The few studies that have done this, such as the limited number available that we reference for Sandy and Harvey, have demonstrated that flooded contaminated sites have in fact redistributed toxins to the surrounding areas. Unfortunately, our findings indicate there will be many more opportunities for such studies to be undertaken in the future.

## Discussion

Taken together, Harvey and Sandy are examples of the risk of contamination due to flooding, a risk that our projections indicate will increase in future climate conditions. Future events do not have to be at the same scale a Harvey and Sandy to realize this risk. Continued sea-level rise due to climate change actually reduces the amount of storm surge needed to cause coastal flooding [39]. Depending on future global emissions rates, sea-level is expected to rise by 20–30 cm by 2050 and between 70–180 cm by 2100 [40]. As a result, globally 250 million people currently live in coastal areas at risk of sea-level rise and annual coastal flooding, by 2050 340 million people will be at risk, and by 2100 630 million people; these estimates are roughly three times greater than previously established figures [41]. Federal, state and city level environmental managers and planners are not blind to these realities [42]. However, even when significant steps have been taken to protect a vulnerable contaminated site, like the San Jacinto River Waste Pits in Houston, they have been flooded [43]. Part of the reason for this can be attributed to budget constraints for effective management, even though billions of dollars are spent every year on remediation [44]; part of the reason may also be attributable to flawed flood zone analysis [12] or miscalculations of the risk of climate change [10]. Our results indicate that environmental managers will have to adjust their current processes and standards to account for future climate conditions if they want to mitigate the increasing risk of contaminants spreading into communities and homes beyond the current levels of ‘acceptable’ risk. Our study highlights the lack of existing data tracking or even modelling toxic flows during flood events, limiting our forecasting to potential risk estimations. Further research employing hydrologic modelling and other techniques is needed to map the pathways and potentially impacted areas from toxic flows from inundated contaminated sites.

The US has one of the most robust environmental regulatory systems of any country, yet we demonstrated that a significant portion of the US population is at risk of contamination due to flooding of toxic sites, despite using only a subset of potentially toxic sites, a subset of the population living in dense urban areas, and a conservative estimate of future atmospheric carbon concentrations. Given the rapid increase in built environment, urbanization, and industrial production globally, much of which is along coastlines or otherwise exposed to flooding, and the global risk of contamination from flooded toxic sites is rapidly increasing [17]. The global burden of disease from toxic pollution already leads to several million early deaths annually [45]. Our findings suggest that under future climate conditions this rate may significantly increase unless remediation, mitigation, and adaptation measures are undertaken immediately to combat both toxic sites and the impacts of climate change.

## Supporting information

S1 Tabulated Data File Summary statistics of data for 2021, 2031, 2041, 2051, and 2061. (XLSX)
