# Correction: Toxic trajectories under future climate conditions

**DOI:** 10.1371/journal.pone.0230094

**Published:** 2020-03-02

**Authors:** Richard A. Marcantonio, Sean Field, Patrick M. Regan

There is an error in the first sentence of the fourth paragraph of the Introduction. The correct sentence is: Additionally, the aggregate risk of flood is often misunderstood.

There is an error in the fifth sentence of the fourth paragraph of the Introduction. The correct sentence is: Understanding the dispersion of toxins from flooding under different potential climate conditions—a rapidly increasing, but minimally accounted for global phenomenon and risk—is essential for effective environmental policy and climate adaptation decision-making [17].

There is an error in the fifth sentence of the first paragraph of the Local level empirical analysis section of the Results and findings. The correct sentence is: More recent events, such as Tropical Storm Imelda that dropped as much rain in parts of Houston as did Hurricane Harvey, are shifting the baseline precipitation distribution of cities such that these storms may eventually cease to be considered outliers.

Fig 1, “Cities with predicted flood probabilities above their historical baseline for the years 2021, 2031, 2041, 2051, and 2061,” is incorrect. Please see the complete, correct Fig 1 here.

**Fig 1:**
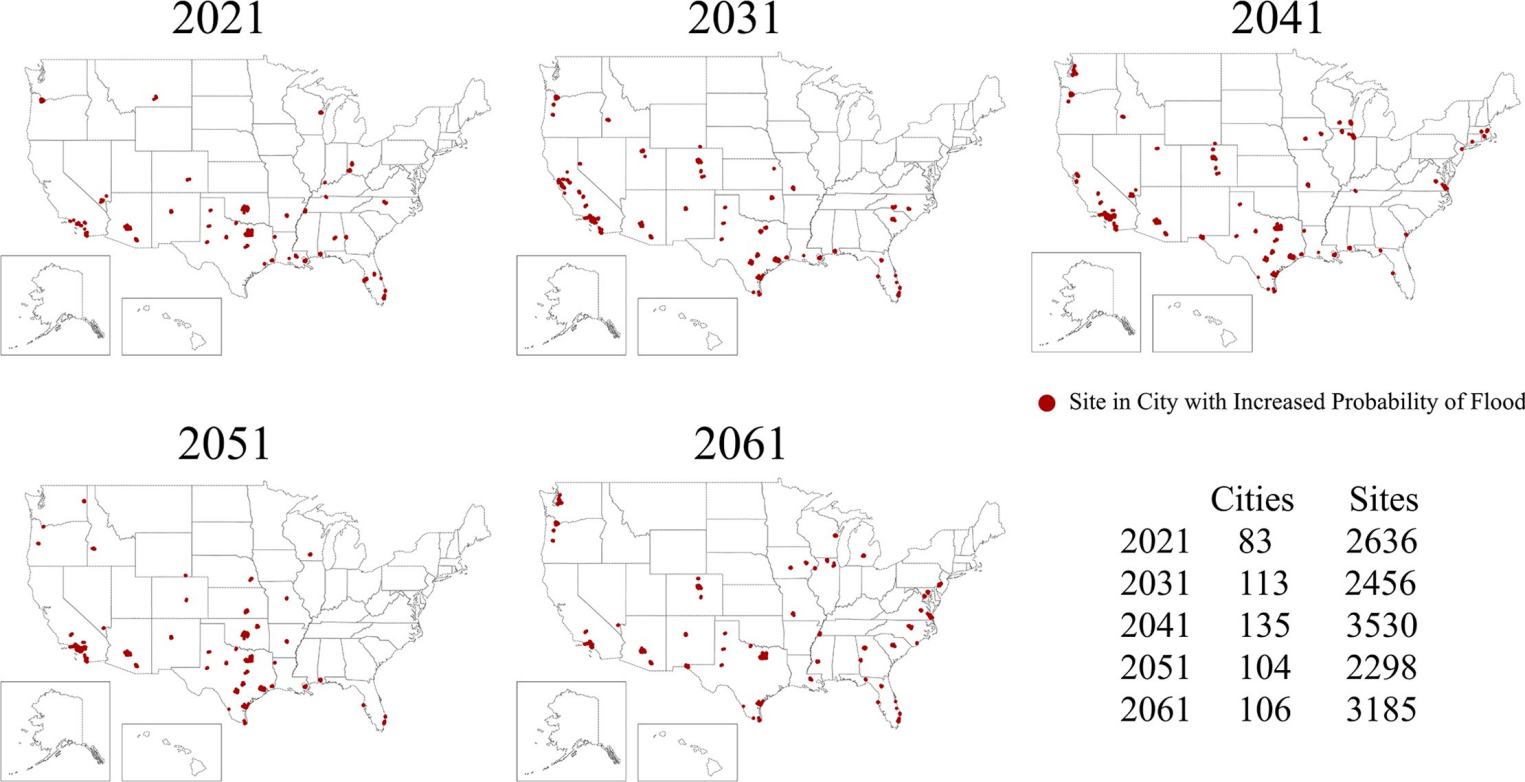
Cities with predicted flood probabilities above their historical baseline for the years 2021, 2031, 2041, 2051, and 2061. The number of sites at risk in each city varies resulting in the possibility of fewer cities, but more sites at risk of flooding—and vice versa—in a given year.
